# Role of Vaginal and Gut Microbiota in Human Papillomavirus (HPV) Progression and Cervical Cancer: A Systematic Review of Microbial Diversity and Probiotic Interventions

**DOI:** 10.7759/cureus.85880

**Published:** 2025-06-12

**Authors:** Hrishikesh D Pai, Rashmi Baid, Nandita P Palshetkar, Rishma Pai, Arnav Pai, Rohan Palshetkar

**Affiliations:** 1 Obstetrics and Gynecology, Lilavati Hospital and Research Centre, Mumbai, IND; 2 Obstetrics and Gynecology, DY Patil IVF Centre, Navi Mumbai, IND

**Keywords:** cervical cancer, cervical intraepithelial neoplasia, human papillomavirus, lactobacillus, microbiome

## Abstract

Cervical cancer remains a major global health concern, primarily associated with persistent infection by high-risk human papillomavirus (HrHPV) types. Both gut and vaginal microbiome may influence the progression from HPV infection to cervical intraepithelial neoplasia (CIN) and cervical cancer.

We performed a systematic review to study the relevant literature on gut and cervical microbiota in patients with HPV infection, CIN, and cervical cancer, as well as the role of probiotics in managing these conditions. The protocol was registered in the PROSPERO database (#CRD42024584685). We searched PubMed, the Cochrane Library, Web of Science, and Google Scholar from their inception to September 2024. Two reviewers independently checked study eligibility. Both reviewers were responsible for data extraction. Disagreements were resolved by a third senior reviewer. The review was conducted in accordance with Preferred Reporting Items for Systematic Reviews and Meta-Analyses (PRISMA) guidelines for reporting systematic reviews. The Joanna Briggs Institute (JBI) checklist was used to assess the risk of bias of the included studies.

In total, 82 papers were included in this review. Microbial dysbiosis plays a significant role in HPV infection, its progression, and clearance. The analysis of the microbiome reveals that the transition from HPV infection to CIN to cervical cancer involves a shift from a *Lactobacillus*-dominated, healthy microbiome to one dominated by pathogenic genera. Women with CIN and cervical cancer demonstrated increased microbial diversity compared to HPV-positive individuals. Pathogenic organisms such as *Gardnerella, Prevotella, Sneathia, Streptococcus,* and *Porphyromonas* were more prevalent in the patient population compared to controls. Probiotics were effective in restoring vaginal microbiota and managing HPV clearance, and were also associated with cytological and inflammatory improvement rates.

Patients with HPV, CIN, and cervical cancer exhibited a microbial community characterised by an increased abundance of pathogenic genera and reduced levels of beneficial *Lactobacillus* species. Probiotics could be used as a prophylactic or an adjuvant therapy while treating HPV infection, CIN, and cervical cancer.

## Introduction and background

Cervical cancer is a major global public health concern and is primarily associated with persistent infection by high-risk human papillomavirus (hrHPV) types [1]. It is the fourth most prevalent cancer among women worldwide [2]. Globally, in 2022, approximately 6,60,000 new cases and around 3,50,000 deaths were reported [1]. According to the World Health Organisation (WHO), cervical cancer is more prevalent in low- and middle-income countries (LMIC), owing to the lack of vaccination and screening facilities, accounting for over 90% of deaths worldwide [3].

The risk factors for developing cervical cancer include human papillomavirus (HPV) infection, smoking, low socio-economic status, early marriage, young age at first coitus, multiple sexual partners, and multiple childbirths [4]. The hrHPV types associated with cervical cancer are HPV 16 and 18. Other high-risk types, including HPV 31, 33, 35, 39, 45, 51, 52, 56, 58, and 59, are implicated in a smaller percentage of cervical cancers, but remain strongly linked to cervical lesions [5]. HPV infection is the risk factor in 99.7% of cervical cancer cases [6].

The progression from HPV infection to cervical cancer often involves intermediate stages, such as cervical intraepithelial neoplasia (CIN). CIN refers to precancerous cervical lesions resulting from persistent infection with hrHPV types. The CIN classification system is divided into three grades: CIN 1, CIN 2, and CIN 3, each indicating a progressively higher level of cellular abnormality. CIN 1 lesions are often self-limiting and low risk, while CIN 2 and CIN 3 warrant closer monitoring and potential intervention due to their higher likelihood of progression to invasive cancer [7]. CIN 1 and CIN 2 clearance is possible during the early stages. A 38% clearance of CIN 2 has been reported at one year, and a 68% clearance at three years [8]. The gradual progress offers a window of opportunity for intervention before late-stage disease sets in. Countries around the world are working towards eradicating cervical cancer by 2030 through vaccination, early detection, intervention, and education on other risk factors like smoking [1].

Emerging evidence suggests that microbiota, both gut and vaginal, may influence progression from HPV infection to CIN and cervical cancer [9]. The gut microbiota is critical in digestion, metabolism, and immune function. A healthy gut microbiome supports homeostasis and overall health, while dysbiosis, or an imbalance in microbial composition, is associated with diseases, including cancer. Recent studies highlight an association between gut microbiota composition and cervical cancer risk [10]. The vaginal microbiota is predominantly composed of *Lactobacillus* species in healthy women. A healthy vaginal microbiome maintains an acidic environment that inhibits pathogenic organisms through a balance of *Lactobacillus* species. Conversely, dysbiosis increases susceptibility to infections, including HPV [11].

This systematic review focused on the diversity and composition of gut and cervicovaginal microbiota in women diagnosed with HPV infection, CIN, and cervical cancer. It also explored the potential role of probiotics in managing these conditions.

## Review

Methodology

Protocol and Registration

This systematic review adheres to the relevant criteria of the Preferred Reporting Items for Systematic Reviews and Meta-analyses (PRISMA) statement [12] and is registered on PROSPERO (#CRD42024584685).

Eligibility Criteria

All studies investigating gut or vaginal microbiota in women with HPV infection, CIN, and cervical cancer were included with no restrictions on age or ethnicity. Studies using 16S rRNA gene sequencing or whole genome metagenomic sequencing techniques only were included. Studies using alternative microbiome assay techniques were excluded. In studies where both 16S rRNA gene sequencing and whole genome metagenomic sequencing were used, the data obtained through the 16S rRNA method were prioritized. Original research articles (cross-sectional studies, cohort studies, and randomised controlled trials (RCTs)) reporting in the English language were included.

Animal studies, editorials, case series, case reports, conference proceedings, abstract-only papers, secondary data analysis, and studies with fewer than 15 participants were excluded.

Information Sources and Search Strategy

A systematic search was conducted across PubMed, Cochrane Library, Google Scholar, and Web of Science from inception to 30th September 2024. The initial search was performed using generic and medical subject heading (MeSH) terms for "probiotic, prebiotic, lactobacillus, lactobacilli, acidophilus, Bifidobacterium, enterococcus, saccharomyces, VSL#3, fructan, oligofructose, fructose, fructooligosaccharide, boulardii, microbiome, microbiota, intestinal flora, microbiomes, microbiotas, dysbiosis, microflora, gut, intestine, intestinal, alimentary, bowel, digestive, enteric, gastrointestinal, genital, vagina, CIN, cervical intraepithelial neoplasia, cervical cancer, cervical neoplasm, cervix cancer, cancer of the cervix, HPV, human papillomavirus."

From Google Scholar, the first 100 results were cross-referenced with PubMed, Web of Science, and Scopus results to ensure comprehensiveness. References unique to Google Scholar were included for data extraction and quality assessment. Google Scholar search used keywords: "prebiotic OR probiotic OR Vaginal microbiota OR Gut Microbiota and Human Papillomavirus infection OR Cervical intra-epithelial Neoplasia OR Cervical Cancer."

Study Selection and Data Extraction

The initial search results were imported into EndNote X9.3.3 software (Clarivate, Philadelphia, PA, USA) and then uploaded to the JBI SUMARI platform (Joanna Briggs Institute, Adelaide, SA, Australia) [13]. Two reviewers separately screened titles, abstracts, and full-text articles to confirm their eligibility. These studies assessed gut and vaginal microbiota diversity and composition in women with HPV infection, CIN, or cervical cancer without date restrictions. Baseline data regarding study characteristics, population demographics, and microbiome diversity were extracted. Disagreements, if any, were resolved by a third reviewer who acted as an adjudicator.

These data from cross-sectional and cohort studies were separately reported. The extracted data was categorized into several categories: (i) study characteristics (author, year of publication, country); (ii) participant characteristics (sample size, mean age, disease condition, HPV type, specimen used); (iii) microbiome assay; (iv) specific bacterial taxonomy associated with the disease; (v) type of microbiome-disease association; (vi) intervention used; and (vii) other relevant conclusions. The combined means formula was applied when means were reported for multiple subgroups [14]. The combined mean formula is as follows:

\[ \text{Mean} = \frac{N_1 M_1 + N_2 M_2}{N_1 + N_2} \]

Where N represents the sample size, M represents the mean, and subscripts 1 and 2 indicate the two subgroups.

Quality Assessment

The JBI assessment tools were used to evaluate the risk of bias. This assessment aimed to evaluate the methodological quality of the studies and to identify possible sources of bias in their design, execution, and analysis. The assessment was conducted using the JBI SUMARI tool. Separate sets of assessment questions were applied for cross-sectional studies, cohort studies, and RCTs. The adequacy of demographics, procedures, methods of analysis, and confounding factors was considered in this process. Any differences in opinion between the reviewers were resolved by involving a third reviewer for discussion. Detailed information on the methodological assessment can be found in Appendices A-C.

Results

This systematic review explores the status of gut and vaginal microbiome in patients with HPV, CIN, and cervical cancer. It also investigates the potential role of probiotics in cervical cancer prevention. Additionally, it explores the changes in microbiota profiles in the gut and vaginal environments during cervical cancer progression and treatment.

PRISMA Chart

**Figure 1:**
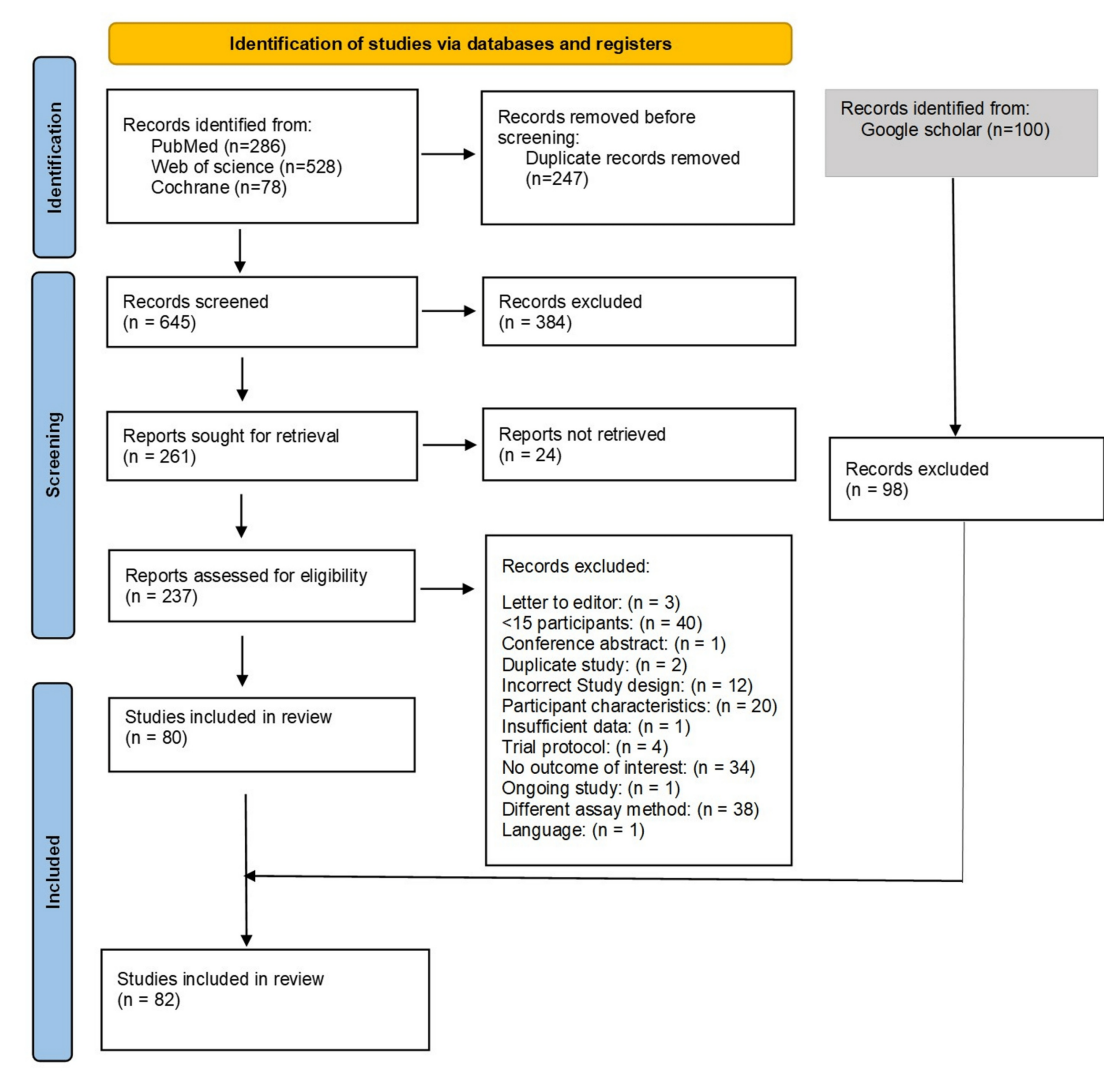
PRISMA flow chart PRISMA: Preferred Reporting Items for Systematic Reviews and Meta-Analyses

After removing duplicates, 645 articles were screened, and 237 were assessed for eligibility. Following the full-text screening, 80 studies were included from PubMed, Web of Science, and Cochrane. Two additional papers from Google Scholar were also included. A total of 82 papers were included in this review. These included 57 case-control studies, 21 cohort studies, and four RCTs. Figure 1 illustrates the PRISMA flow chart.

Study Characteristics

The majority of the 82 included studies were conducted in China (52.4%), followed by the United States of America (7.30%), Republic of Korea, Italy and Mexico (4.87% each), Ethiopia, Kingdom of the Netherlands and Poland (3.65% each), Russia, Canada, South Africa, and Sweden (2.40% each), United Kingdom of Great Britain and Northern Ireland, Spain, Colombia, Costa Rica, Greece, Belgium and Ethiopia (1.20% each). Most studies focused on the vaginal microbiome, and only three studies assessed rectal samples [15-17]. Table 1 summarises studies assessing gut and cervicovaginal microbiota, and Table 2 focuses on the effects of probiotic supplements. Dong et al. evaluated vaginal, rectal swabs and urethral, along with cervical cell-shedding fluid. They found that HPV-positive women exhibited lower Firmicutes levels and elevated Actinobacteriota and Proteobacteria, except in the urethra. Statistical analysis revealed significant differences in *Fastidiosipila* (cervix), *Lactobacillus* (vagina), and *Ruminococcus* (rectum) [18]. Other studies reported microbiome data primarily from vaginal samples.

**Table 1:** Characteristics of studies assessing gut and cervico-vaginal microbiota in women with HPV infection, CIN, and cervical cancer. A: Vaginal samples;B:Cervical swabs; C: Rectal swabs;D: Cervicovaginal swab; HPV: Human Papillomavirus; CIN: Cervical intraepithelial neoplasia; CC: Cervical cancer; LACC: Locally advanced cervical cancer; SCC: Squamous cell carcinoma; HCW: Healthcare worker

First author	Country	No. of cases	No. of controls	HPV sub-types	CIN sub-tyes	Cancer stage	Sample type (A, B, C, D)	Sample collected	Method of sequencing	Region of analysis
Sims TT, et al. 2021 [17]	USA	CC: 55	-	-	-	-	C	HCW	16s rRNA	V4
Dong YH, et al. 2024 [18]	China	HPV: 51	HPV -ve: 51	HPV 16, 18, 58, 44, 81	-	-	A, B, C	HCW	16s rRNA	
Yang Q, et al. 2020 [19]	China	HPV: 27	HPV -ve: 25	HPV 16	-	-	A, B	HCW	Shotgun metagenomics proﬁlin	-
Lopez Filloy M, et al. 2020 [20]	Mexico	HPV: 35	HPV -ve: 28	HPV 6, 11, 16, 18, 26, 31, 35, 39, 45, 51, 52, 53, 56, 58, 59, 66, 68, 73, and 82.	-	-	D	HCW	16s rRNA	V4
Liu J, et al. 2020 [21]	China	HPV: 91	Negative control: 31	HPV 16, 18, 56, 52, and 58	-	-	A	HCW	16s rRNA	NM
Lee JE, et al. 2013 [22]	Korea	HPV: 23	HPV -ve: 45	HPV genotypes 16, 18, 33, 35, 39, 45, 51, 52, 56, 58, 59, and 66	-	-	D	HCW	16s rRNA	V2-V3
Zeber-Lubecka N, et al. 2024 [23]	Poland	HPV: 46	Healthy: 27	HPV 16 and 18	-	-	B	HCW	16s rRNA	NA
Onywera H, et al. 2019 [24]	South Africa	HPV: 37	HPV -ve: 50	NA	-	-	B	HCW	16s rRNA	V3-V4
Xia Y, et al. 2022 [25]	China	HPV: 92	HPV -ve: 43	HPV 16, 18, and 12 other types	-	-	-	-	16s rRNA	V3-V4
Chao XP, et al. 2019 [26]	China	HPV: 65	HPV -ve: 86	-	-	-	A	HCW	16s rRNA	V4
Xu XL, et al. 2022 [27]	China	HPV: 230	HPV -ve: 193	-	-	-	A, B	HCW	16s rRNA	V3-V4
Wei ZT, et al. 2021 [28]	China	HPV: 30	HPV -ve: 30	-	-	-	A, B	HCW	16s rRNA	V3-V4
Liu S, et al. 2022 [29]	China	HPV: 53	HPV -ve: 16	HPV 16, 52, 33, and 58	-	-	A	-	16s rRNA	V4
Mei L, et al. 2022 [30]	China	HPV: 58	HPV -ve: 42	HPV 16, 18, 31, 33, 35, 39, 45, 51, 52, 53, 56, 58, 59, 66, 68, 73 and 82	-	-	A	-	16s rRNA	V3-V4
Fang B, et al. 2023 [31]	China	HPV: 20	HPV -ve: 20	-	-	-	B	HCW	16s rRNA	V3-V4
Wang TT, et al. 2024 [32]	China	HPV: 150	HPV -ve: 35	HPV 52, 58, 16	-	-	A, B	HCW	16s rDNA	V3-V4
So KA, et al. 2024 [33]	S.Korea	HPV: 75	-	-	-	-	D	HCW	16s rRNA	V3-V4
Zhou YY, et al. 2019 [34]	China	HPV: 42	HPV -ve: 20	-	-	-	A	HCW	16s rRNA	V3-V4
Hu JT, et al. 2022 [35]	China	HPV: 94	HPV -ve: 182	HPV 16, 39, 52, 56, 58	-	-	B	-	16s rRNA	V3-V4
Zeng M, et al. 2023 [36]	China	HPV: 90	HPV -ve: 45	HPV 16, 51, 53, 52	-	-	B		16s rRNA	NA
Dou PN, et al. 2024 [37]	China	HPV: 70	HPV -ve: 136	HPV 52, 16	-	-	A, B	HCW	16s rRNA	V3-V4
Huang XJ, et al. 2018 [38]	China	HPV: 239	HPV -ve: 41	HPV 16, 52, 58	-	-	B	HCW	16s rRNA	V4-V5
Happel AU, et al. 2023 [39]	S.Africa	HPV: 22	No BV: 19	HPV 35, 16, 53, 81, 90, 214	-	-	D	HCW	16S rRNA	V4
Cheng LQ, et al. 2020 [40]	Sweden	HPV: 144	HPV -ve: 133	HPV 56, 45, 52, 31,33,6, 11, 16, 18, 39, 42, 56	-	-	A	HCW	16s rRNA	V3-V4
Usyk M, et al. 2020 [41]	Costa rica	HPV: 273	-	HPV 16, 18, 31, 33, 35, 39, 45, 51, 52, 56, 58, or 59	-	-	D	-	16s rRNA	V4
Camargo M, et al. 2022 [42]	Colombia	HPV: 66	-	-	-	-	B	-	16s rRNA	-
Wang WP, et al. 2023 [43]	China	HPV: 32	-	HPV 16, 18, 52, 51	-	-	A	-	16s rRNA	V3-V4
Di Paola M, et al. 2017 [44]	Italy	HPV: 55	HPV -ve: 17	-	-	-	D	-	16s rRNA	V3-V5
Rodríguez SP, et al. 2022 [45]	Spain	HPV: 21	-	-	-	-	A	-	16s rRNA	V1-V4
Wu RT, et al. 2019 [46]	China	HPV: 90	HPV -ve: 43	-	-	-	B	HCW	16s rRNA	V4
Shannon B, et al. 2017 [47]	Canada	HPV: 23	HPV -ve: 36	-	-	-	D	HCW	16s rRNA	V3-V4
Berggrund M, et al. 2020 [48]	Sweden	HPV: 64	HPV -ve: 32	HPV 16	-	-	A	Self-sampling	16s rRNA	V2-V9 & V3-V4
Shi WP, et al. 2022 [49]	China	HPV: 73	-	HPV 52, 16, 58	-	-	B	-	16s rRNA	V4-V5
Dong B, et al. 2022 [50]	China	HPV: 90	HPV -ve: 30	-	-	-	A	-	16s rRNA	V3-V4
Molina MA, et al. 2023 [51]	Netherlands	HPV: 141	-	-	-	-	B	HCW	CiRNAseq microbiome profiling	-
Molina, M. A, et al. 2024 [52]	Netherlands	HPV: 141	-	-	-	-	B	-	ciRNAseq technology	-
Nieves-RamÃrez ME, et al. 2021 [53]	Mexico	CIN 1-90 and CIN 2&3- 31	No CIN: 107	-	CIN 1, 2, 3	-	A	HCW	16s rRNA	V3
Liu YR, et al. 2023 [54]	China	CIN1: 233, CIN2: 147, CIN3: 33	No CIN: 279	-	CIN 1, 2, 3	-	A	HCW	16s rRNA	V4
Lin S, et al. 2022 [55]	China	CIN1: 4, CIN2: 6, CIN3: 13	No CIN: 60	-	CIN 1, 2, 3	-	A, B	-	16s rRNA	V3-V4
Logel M, et al. 2024 [56]	Canada	CIN1: 50, CIN2: 40, CIN3: 42	No CIN: 54	HPV 16, 18	CIN 1, 2, 3	-	B	HCW	16s rRNA	V3-V4, V5-V6
Lee YH, et al. 2020 [57]	S.Korea	CIN1-: 24, CIN 2+: 42	NA	-	CIN 1-, CIN 2+	-	A	HCW	16s rRNA	V3
Kwasniewski W, et al. 2018 [58]	Poland	HPV +ve: 180	HPV -ve: 70	-	-	-	B	HCW	16s rRNA	V4
Zhang HW, et al. 2018 [59]	China	CIN: 26	-	HPV 16, 18, 31, 33, 35, 39, 45, 51, 52, 56, 58, 59, and 68	CIN 2, 3	-	B	-	16s rRNA	V3-V4
Caselli E, et al. 2020 [60]	Italy	CIN: 85	-	-	CIN 2 ,3	-	D	-	16s rRNA	-
Mitra A, et al. 2021 [61]	UK	CIN: 103	CIN -ve: 39	-	CIN 1, 2, 3	-	D	-	16s rRNA	V1-V3
Mitra A, et al. 2020 [62]	USA	CIN: 87	-	-	CIN 2	-	B	-	16s rRNA	V1-V2
Li YP, et al. 2023 [63]	China	CC: 26	Normal tissue: 53	-	-	-	A	HCW	16s rRNA	V4
Wang ZZ, et al. 2022 [64]	China	LACC: 26	CC -ve: 40	HPV 16, 18, 11, 31, 33, 35, 39, 45, 51, 52, 56, 58, 59, 66, and 68	-	-	A	-	16s rRNA	V3-V4
Manzanares-Leal GL, et al. 2022 [65]	Mexico	CC: 60	CC -ve: 60	-	-	-	D	-	16s rRNA	-
Zeber-Lubecka N, et al. 2022 [66]	Poland	CC: 16	CC -ve: 30	-	-	SCC	B	-	16s rRNA	-
Wang Y, et al. 2024 [67]	China	CC: 125	-	-	-	IB-IVB	A	-	16s rRNA	V3
Manzanares-Leal GL, et al. 2021 [68]	Mexico	CC: 68	-	-	-	IB2-IVA	C	-	16s rRNA	-
Yang Y, et al. 2024 [69]	China	CC: 20, HPV: 20	HPV -ve: 19	-	-	-	A, B	HCW	16s rRNA	V4
Fan Z, et al. 2024 [70]	China	HPV: 40; CIN: 40; CC: 18	HPV -ve: 27	HPV 16, 18	-	-	A	HCW	16s rRNA`	V4
Liu H, et al. 2022 [71]	China	HPV: 34; CIN: 40; CC: 41	-	-	-	-	B	-	Illumina HiSeq 2500 platform	-
Oh HY, et al. 2015 [72]	Korea	CIN: 70	CIN -ve: 50	-	-	-	B	HCW	16s rRNA	V1-V3
Piyathilake CJ, et al. 2016 [73]	USA	CIN & HPV: 430	-	HPV 16, 18, 31, 33, 35, 39, 45, 51, 52, 56, 58, 59, and 68	CIN 1, 2, 3	-	B	HCW	16s rRNA	V4
Teka B, et al. 2023 [74]	Ethiopia	CC: 60; CIN: 25	CIN -ve: 35	-	-	-	B	HCW	16s rRNA	V4
Guo C, et al. 2022 [75]	China	HPV: 119	HPV -ve: 30	-	-	-	A, B	HCW	16s rRNA	V4-V5
Ivanov KM, et al. 2023 [76]	Russia	CIN: 46; CC: 17	CIN, CC -ve: 77	-	-	-	D	-	16s rRNA	V3-V4
Cheng WY, et al. 2020 [77]	China	HPV: 26; CIN: 40; CC: 32	HPV -ve: 33	-	-	-	A	HCW	16s rRNA	V4
Zhang Y, et al. 2024 [78]	China	HPV: 25	HPV -ve: 17	-	-	-	D	HCW	16s rRNA	V3-V4
Zhang CT, et al. 2018 [79]	China	CIN1: 62, CIN2: 19, CIN3: 21	CIN -ve: 64	-	CIN 1, 2, 3	-	B	HCW	16s rRNA	V3-V4
Sofou E, et al. 2023 [80]	Greece	HPV +ve; CIN: 877	-	HPV 16, 18, and others	CIN 1, 2, 3	-	A, B	Self-collected and doctor-collected	16s rRNA	V3-V4
Li XG, et al. 2023 [81]	China	CC: 80; CIN: 80; HPV: 80	HPV -ve: 80	-	-	-	A	-	16s rDNA	V3-V4
Molina MA, et al. 2022 [82]	Netherlands	HPV: 100; CIN: 200	HPV -ve: 44	HPV 16, 18, 31, 33, 35, 39, 45, 51, 52, 56, 58, 59, 66, 68, and 73	-	-	B	-	16S and 18S rRNA genes	-
Zhai QZ, et al. 2021 [83]	China	HPV: 29; CIN: 72; CC: 38	HPV -ve: 29	-	-	-	B	-	16s rRNA	V3-V4
Peremykina A, et al. 2024 [84]	Russia	CIN: 103	HPV -ve: 22	-	-	-	B	-	16s rRNA	V4
Zhang W, et al. 2024 [85]	China	HPV: 21; CC: 22	HPV -ve: 22	HPV 16, 18, 58, 33, 51, 52, 53, 68.	-	-	A	-	16s rRNA	-
Chao XP, et al. 2021 [86]	China	CIN: 83 HPV: 86	HPV -ve: 103	HPV 16 and 18 + other types	CIN 2	-	A	HCW	16s rRNA	V4
Chao XP, et al. 2020 [87]	China	HPV: 198	HPV -ve: 131	HPV 16 and 18	CIN 1, 2	-	A	-	16s rRNA	V4
Zhang YC, et al. 2022 [88]	China	HPV: 159; CIN: 84	HPV -ve: 113	HPV 16, 52, 58	CIN 1, 2, 3	-	A, B	-	16s rRNA	-
Zheng WH, et al. 2023 [89]	China	CIN: 30; CC: 15	HPV -ve: 15	-	CIN 1, 2, 3	-	A	HCW	16s rRNA	V3-V4
Ma YY, et al. 2023 [90]	China	CC: 27; CIN: 81; HPV: 22	HPV -ve: 30	-	-	-	A	-	16s rRNA	V4
McKee KS, et al. 2020 [91]	USA	CIN: 109; HPV: 110	HPV -ve: 89	-	-	-	A	-	16s rRNA	V4

**Table 2:** Characteristics of studies assessing the effect of probiotic supplementation. A:Vaginal samples; B: Cervical swab; HPV: Human Papillomavirus; LSIL: Low-grade squamous intraepithelial lesion

First author	Country	Study design	No. of cases	No. of controls	HPV sub-types	Sample type	Method of sequencing	Region of analysis
Liu Y, et al. 2024 [92]	China	Prospective controlled pilot study	HPV: 50	50	-	A	16 srRNA	-
DI Pierro F, et al. 2021 [93]	Italy	Observational, open, pilot study	HPV: 35	-	HPV 16, 18, 66, 68, 58, 45, 53, 51, 52, 35	A	16 srRNA	V3
Ou YC, et al. 2019 [94]	China	Randomized placebo-controlled trial	HPV: 62	HPV: 59	-	B	-	-
Verhoeven V, et al. 2013 [95]	Belgium	Prospective controlled pilot study	HPV: 24	LSIL: 27	HPV 16, 18, 31, 33, 35, 39, 51, 52, 53, 56, 58, 59, 66, and 68	B	-	-
Dellino M, et al. 2020 [96]	Italy	Pilot study	HPV: 80	HPV: 80	HPV 16, 51, 42, 35, 18, 52, 68	B	-	-

Human Papillomavirus (HPV)

Microbiome data from 23 cross-sectional studies of HPV-infected women were analysed [19-40]. Several phyla correlated with HPV infection, including Actinobacteria, Firmicutes, and Fusobacteria. Specific genera like *Sneathia*, *Gardnerella, Atopobium, Prevotella*, and *Megasphaera* were more commonly associated with HPV-positive individuals. Conversely, genera like *Lactobacillus* and other Firmicutes were more abundant in controls, reflecting a healthier microbiota in HPV-negative individuals. Certain species, like *G. vaginalis* and *L. iners*, were more common in HPV-positive individuals, implying that *L. iners* lacks the protective properties of other *Lactobacillus* species against HPV infection. *Limosilactobacillus mucosae* was found to be more frequent in HPV-negative women.

Among the cohort studies, 12 studies investigated the vaginal microbiome in HPV patients during follow-up periods ranging from 21 days to 24 months [41-52]. HPV-positive women exhibited distinct microbiomes compared to controls, with differences in diversity, species composition, and genera linked to HPV infection, persistence, clearance, and progression.

In HPV positive women, *G. vaginalis *was associated with CIN2+ progression (cervical dysplasia), while *L. iners* was correlated with HPV clearance. Women with higher viral load (VL) exhibit an increased abundance of *Acinetobacter*, *Megasphaera*, and *Sneathia*, whereas *L. iners* is found in higher amounts in women with low VL. During follow-up, species like *L. crispatus* and *L. gasseri* were linked to HPV clearance, while *Prevotella* and *Gardnerella* were associated with HPV progression.

At the 12- and 24-month follow-ups, *Lactobacillus* spp. were associated with HPV clearance, while genera such as *Sneathia, Prevotella*, and *Megasphaera* were linked to disease progression and persistence. Firmicutes, Proteobacteria, and Actinobacteria were notably associated with HPV status, with *Lactobacillus* species dominant in HPV-negative controls.

Cervical Intraepithelial Neoplasia (CIN)

Six cross-sectional studies analysed microbiome data in CIN patients [53-58]. The microbiome in women with CIN differed from healthy controls, though diversity findings were inconsistent. Phyla such as Actinobacteria, Bacteroidetes, and Fusobacteria were linked to CIN, while Firmicutes were notably reduced in CIN3. Genera like *Gardnerella* and *Prevotella* were associated with CIN. It was observed that *L. iners* exhibited dual roles, being both protective and harmful in CIN patients. The overall microbial community structure differed, with higher levels of *Lactobacillus* in the control group and a more diverse range of bacteria in CIN, including species like *G. vaginalis, Actinomyces turicensis*, and *Mycoplasma hominis*.

Four cohort studies assessed microbiome changes in CIN patients [59-62]. A significant shift in diversity and species occurred in the vaginal microbiome of CIN patients, spanning CIN 1, CIN 2, and CIN 3 at different stages of disease and treatment. Species richness and microbiota diversity were greater in pre-treatment groups than in post-treatment groups. *Lactobacillus* spp., particularly *L. iners*, were prevalent in CIN 2, while *G. vaginalis, A. vaginae*, and *Ureaplasma parvum* dominated in CIN 3. Post-treatment, microbiomes were enriched with bacterial vaginosis (BV)-associated taxa, including BVAB2, *P. bivia*, and *Veillonellaceae*, while controls had more abundant *L. crispatus*. At 12- and 24-month follow-ups, diversity differences between CIN regressors and non-regressors were minimal, though non-regressors tended to exhibit higher diversity. *Lactobacillus* spp. were linked to disease regression, while genera like *Prevotella, Megasphaera, Sneathia,* and *Atopobium* were associated with non-regression.

Cervical Cancer

Five studies analyzed microbiome data in cervical cancer patients, including two studies on rectal samples [15,16,63-65]. Phyla like Bacteroidetes, Firmicutes, Proteobacteria, Actinobacteria, Fusobacteria, and Tenericutes have been associated with cervical cancer. Genera specifically associated with the disease included *Nocardioides*, *Lachnoanaerobaculum*, *Clostridium sensu stricto 1, Ruminococcaceae UCG_014, *and* Jonquetella*. Genera such as *Prevotella, Porphyromonas*, and *Dialister* indicated altered microbial environments in affected individuals [15,16].

Three studies analyzing cervical samples [63-65] found that the viral families *Anelloviridae* and *Papillomaviridae* correlated with cervical cancer. Genera such as *Streptococcus, Peptostreptococcus, Enterococcus, Escherichia-Shigella, Staphylococcus*, and *Klebsiella* were overrepresented in cancer patients compared to controls. Specific genera, such as *Corynebacterium* and *Staphylococcus*, were similarly linked to the disease.

Among four cohort studies [17,66-68], Sims et al. [17] evaluated rectal samples, while the remaining studies focused on vaginal samples. Gut microbiome diversity decreased after chemoradiation treatment (CRT). Short-term survivors had enriched levels of genera such as *Porphyromonas, Porphyromonadaceae,* and *Dialister*, whereas *Escherichia-Shigella, Enterobacteriaceae,* and *Enterobacteriales* were associated with better long-term survival.

At baseline, cervical cancer patients had higher vaginal microbiome diversity compared to controls, with genera such as *Streptococcus, Prevotella, Fusobacterium,* and *Peptoniphilus* positively associated with the disease, while *Lactobacillus* was negatively correlated. Following CRT and concurrent chemoradiotherapy (CCRT), bacterial diversity decreased, with Proteobacteria, *Mycobacterium*, and *Serratia* being identified as disease-associated taxa. Chemotherapy and radiotherapy treatments led to the dominance of *S. epidermidis* and *E. faecalis*.

Comparison of Vaginal Microbiome Between the Groups

Twenty-three cross-sectional studies evaluated differences in vaginal microbiomes across HPV-positive, CIN, cervical cancer, and control groups [69-91]. Distinct differences were observed across these patient groups.

HPV-positive individuals exhibited reduced microbiome diversity and species richness. Common phyla included Actinobacteria, Firmicutes, Bacteroidetes, and Tenericutes, dominated by genera such as *Lactobacillus, Gardnerella, *and* Prevotella*, and species like *L. iners, G. vaginalis, L. gasseri, *and* L. crispatus*.

Women with CIN and cervical cancer demonstrated increased microbial diversity compared to HPV-positive individuals. *Gardnerella, Streptococcus, *and* Porphyromonas *were more prevalent in CIN. *Lactobacillus* abundance decreased progressively, particularly in cervical cancer patients. Cervical cancer patients had higher levels of *Prevotella, Bacteroides, Gardnerella, Dialister, *and* Porphyromonas*. In contrast, controls exhibited a predominance of *Lactobacillus* (Firmicutes phylum) and a more stable microbiome with greater diversity and richness. These findings indicate that HPV infection, CIN, and cervical cancer are linked to increased microbial diversity, driven by pathogenic genera, whereas healthy controls maintain a stable, *Lactobacillus*-dominated microbiome.

Probiotics in the Treatment for HPV, CIN, and Cervical Cancer

Several studies have evaluated the effects of probiotic therapy on vaginal microbiota in patients with HPV, CIN, and cervical cancer.

Improvement in Vaginal Microbiota

Liu et al. (2024) found that in hrHPV patients, probiotic treatment significantly increased the abundance of Firmicutes (the phylum containing *Lactobacillus*) while reducing Bacteroidetes and Actinobacteria levels. At the genus level, *Lactobacillus* abundance increased significantly, while *Prevotella* and *Gardnerella* decreased, indicating a shift toward a healthier, *Lactobacillus*-dominant microbiota profile [92].

Di Pierro et al. reported that at baseline, 69% of participants exhibited poor *Lactobacillus* content (CST IV). After 90 days of oral *L. crispatus *M247, 97% showed a *Lactobacillus*-dominated microbiota, with 33 participants dominated specifically by *L. crispatus* (CST I). The bacterial richness (as measured by Operational Taxonomic Unit, OTU) decreased significantly from 35±9 to 18±5 post-treatment [93]. Ou YC et al. examined the effects of a combination of *L. rhamnosus *GR-1 and *L. reuteri*. The study group showed significant cytological improvements, including reductions in mildly abnormal cytology such as atypical squamous cells of undetermined significance (ASCUS) and low-grade squamous intraepithelial lesions (LSILs) [94].

Reduction of HPV VL and Enhanced HPV Clearance Rate

A randomized controlled study by Liu et al. reported a 12.13% higher HPV clearance rate in patients treated vaginally for five months with *L. crispatus* Chen-01 compared to the placebo group [92]. Di Pierro et al. observed that 71% of HPV-positive patients at baseline tested HPV-negative after 90 days of oral *L. crispatus* M247 administration [93]. Verhoeven et al. used Yakult (containing​​​ *L. casei *strain Shirota) as the probiotic of choice, reporting clearance rates of 25% at three months and 29.2% at six months, compared to 7.7% and 19.2% clearance in the placebo group [95].

Improvement in Cytology and Inflammatory Status

Verhoeven et al. reported that 50% of cytological abnormalities resolved in the probiotic group, compared to 29.6% in the control group [95]. Liu et al. observed significantly higher rates of cytological and inflammatory improvement in the *L. crispatus* Chen-01 group (82.14% and 77.78%) than in the placebo group (34.62% and 27.27%) [92]. Dellino et al. reported significant resolution of cytological abnormalities within 12 months of oral administration of *L. crispatus* M247 [96].

Safety

Di Pierro et al. noted mild side effects, such as meteorism and flatulence, associated with the oral use of *L. crispatus* M247, which resolved within two weeks [93]. Liu et al. reported no adverse events with vaginal use of *L. crispatus *Chen-01, suggesting it safe and well tolerated [92].

Discussions 

This systematic review explored the vaginal and gut microbiota in patients with HPV, CIN, and cervical cancer. Altered vaginal microbiota plays a significant role in HPV infection, its progression, and clearance. The analysis of the microbiome reveals that the transition from HPV infection to CIN to cervical cancer involves a shift from a *Lactobacillus*-dominated, healthy microbiome to one dominated by pathogenic genera, potentially contributing to disease persistence and progression.

*Lactobacillus* species are abundant in healthy controls and act as protective elements of the cervical and vaginal microbiota. Several protective mechanisms have been identified. One such mechanism is *Lactobacillus* adherence to vaginal and cervical epithelium outcompeting harmful organisms for nutrients. Additionally, *Lactobacilli* help maintain vaginal acidity by producing lactic acid, and secreting anti-microbial compounds like hydrogen peroxide [97]. Dysbiosis reduces *Lactobacillus* abundance while increasing facultative anaerobes, thereby. Enzymes like sialidase, produced by dysbiotic bacteria, disrupt the mucus barrier and damage the cervicovaginal epithelium, making basal cells more susceptible to HPV infections. Furthermore, toxins produced by these bacteria damage host DNA, facilitating the integration of viral oncogenes into the host genome [98].

HPV infection occurs when the virus enters cells through minor injuries or abrasions, replicates, and synthesizes progeny virions, which alter immune responses and damage DNA [99]. HPV-induced carcinogenesis is primarily driven by the viral proteins E6 and E7. E1 and E2 proteins mediate uncontrolled viral replication and influence transcription [100]. The interaction of all mechanisms leads to the enhancement of E6 and E7 genes, enabling immune evasion and persistent infection. The expression of E6/E7 facilitates viral integration, while genetic modification in both viral and host genomes and immunosuppression lead to carcinogenesis [101].

More than 100 HPV subtypes have been identified, with only a subset linked to cervical dysplasia and carcinoma. Persistent HPV infection is a key factor in the progression from CIN to carcinoma. Globally, HPV 16 and 18 infections are responsible for 55-60% and 10-15% of cervical cancers, respectively. Conditions like immunosuppression, smoking, and HIV infection favour HPV persistence and CIN development. The progression from CIN-1 to CIN-2 and CIN-3 is influenced by multiple factors. It can take several years for CIN-2 and CIN-3 to develop into cervical cancer [102].

Cytokines play a central role in viral infection-associated cancers by promoting inflammation and tumour growth. Research has indicated that cytokine deregulation and viral oncoproteins influence the development of cervical precancerous lesions (LSIL and high-grade squamous intraepithelial lesions (HSIL)), advancing to malignant growth, further invasion, and finally, metastasis [103]. Factors such as persisting infection, viral oncogene expression, and instability of the host genome contribute to neoplastic changes. HPV-mediated carcinogenesis involves complex host-viral interactions [101]. A correlation between microbial communities and HPV infection has been stated in several studies, but causal relationships remain difficult to establish.

This study suggests that probiotics, especially those containing *Lactobacillus* strains, are effective in restoring vaginal microbiota and managing HPV infection, progression and clearance. A healthy, *Lactobacillus*-dominant microbiota correlates with high HPV clearance and a lower risk of HPV persistence and infection. Probiotics reverse cytological abnormalities and inflammation, enhance immune responses, and reduce secondary infection risks [104]. In vitro studies demonstrate that *L. gasseri* and *L. crispatus* have cytotoxic effects on HeLa cells [105]. Vaginal probiotics may facilitate a quicker restoration of the vaginal microbiome as an adjuvant with radiotherapy, chemotherapy, or antibiotics use, and oral supplements could help prevent recurrent infections [104].

The limitation of this study includes fewer studies on gut microbiome and methodological differences across the included studies. Although most studies used whole genome sequencing or 16S rRNA, variability in variable regions analysed, pipelines, databases, and cut-offs likely influenced results. Also, confounding factors such as ethnic differences, genetics, and immune characteristics were not systematically accounted for.

## Conclusions

The study concludes that the microbiome plays a crucial role in the progression and response to HPV infection, CIN, and cervical cancer. HPV-positive individuals exhibited a microbial community characterised by an increased abundance of pathogenic genera (*Gardnerella, Sneathia, Prevotella, Megasphaera*) and reduced levels of beneficial *Lactobacillus* species. This dysbiosis may increase the risk of HPV persistence and raise the risk of cervical dysplasia and cancer. The progression from HPV infection to cervical cancer can take many years to develop; hence, early diagnosis and effective intervention are critical in reducing disease burden. Probiotics, particularly those containing *Lactobacillus* strains, are identified as promising preventive and adjuvant therapies for HPV infection and cervical cancer. However, further research is needed to elucidate microbial shifts and their interactions with genetic predisposition, environmental exposures, and immune responses, as well as to focus on the gut microbiome.

## Appendices

Appendix A 

**Table 3:** Critical appraisal of eligible analytical cross-sectional study.

Citation	Ref	Q1	Q2	Q3	Q4	Q5	Q6	Q7	Q8
Yang Q, et al. 2020.	[19]	Y	Y	Y	Y	N	N	Y	Y
Yang Y, et al 2024.	[69]	Y	Y	Y	Y	N	N	Y	Y
Li YP, et al. 2023.	[63]	Y	Y	Y	Y	N	N	Y	Y
Lopez-Filloy M, et al. 2022.	[20]	Y	Y	Y	Y	Y	N	Y	Y
Fan Z, et al. 2024.	[70]	Y	Y	Y	Y	N	N	Y	Y
Liu H, et al. 2022.	[71]	Y	Y	Y	Y	Y	N	Y	Y
Liu J, et al. 2020.	[21]	Y	Y	Y	Y	N	N	Y	Y
Lee JE, et al. 2013.	[22]	Y	Y	Y	Y	Y	Y	Y	Y
Oh HY, et al. 2015.	[72]	Y	Y	Y	Y	Y	Y	Y	Y
Piyathilake CJ, et al. 2016.	[73]	Y	Y	Y	Y	N	N	Y	Y
Zeber-Lubecka N, et al. 2024.	[23]	Y	Y	Y	Y	Y	N	Y	Y
Onywera H, et al. 2019.	[24]	Y	Y	Y	Y	Y	U	Y	Y
Nieves-RamÃ­rez ME, et al. 2021.	[53]	Y	N	Y	Y	Y	U	Y	Y
Happel AU, et al. 2023.	[39]	U	Y	Y	Y	Y	N	Y	Y
Huang XJ, et al. 2018.	[38]	Y	Y	Y	Y	N	N	Y	Y
Teka B, et al. 2023.	[74]	Y	Y	Y	Y	Y	N	Y	Y
Guo CL, et al. 2022.	[75]	Y	Y	Y	Y	Y	Y	Y	Y
Ivanov MK, et al. 2023.	[76]	Y	Y	Y	Y	N	N	Y	Y
Dong YH, et al. 2024.	[18]	Y	Y	Y	Y	N	N	Y	Y
Xia Y, et al. 2022.	[25]	Y	Y	Y	Y	Y	N	Y	Y
Liu YR, et al. 2023.	[54]	Y	Y	Y	Y	Y	U	Y	Y
Cheng WY, et al. 2020.	[77]	Y	Y	Y	Y	N	N	Y	Y
Chao XP, et al. 2019.	[26]	Y	Y	Y	Y	Y	N	Y	Y
Xu XL, et al. 2022.	[27]	Y	Y	Y	Y	N	N	Y	Y
Wei ZT, et al. 2021.	[28]	Y	Y	Y	Y	N	N	Y	Y
Zhang CT, et al. 2018.	[79]	Y	Y	Y	Y	Y	N	Y	Y
Biegert G, et al. 2021.	[15]	Y	Y	Y	Y	Y	N	Y	Y
Liu S, et al. 2022.	[29]	Y	Y	Y	Y	Y	N	Y	Y
Wang ZZ, et al. 2022.	[62]	Y	Y	Y	Y	Y	N	Y	Y
Mei L, et al. 2022.	[30]	Y	Y	Y	Y	N	N	Y	Y
Fang B, et al. 2022.	[31]	Y	Y	Y	Y	Y	N	Y	Y
Lin S, et al. 2022.	[55]	Y	Y	Y	Y	Y	N	Y	Y
Zhang Y, et al. 2024.	[78]	Y	Y	Y	Y	Y	N	Y	Y
Sims TT, et al. 2019.	[16]	Y	Y	Y	Y	Y	Y	Y	Y
Sofou E, et al. 2023.	[80]	Y	Y	Y	Y	Y	N	Y	Y
Wang TT, et al. 2024.	[32]	Y	Y	Y	Y	Y	N	Y	Y
Li XG, et al. 2023.	[81]	Y	Y	Y	Y	Y	N	Y	Y
So KA, et al. 2024.	[33]	Y	Y	Y	Y	N	N	Y	Y
Molina MA, et al. 2022.	[82]	Y	Y	Y	Y	Y	N	Y	Y
Zhai QZ, et al. 2021.	[83]	Y	Y	Y	Y	N	N	Y	Y
Peremykina A, et al. 2024.	[84]	Y	Y	Y	Y	N	N	Y	Y
Kwasniewski W, et al. 2018.	[58]	Y	Y	Y	Y	Y	Y	Y	Y
Zhou YY, et al. 2019.	[34]	Y	Y	Y	Y	N	N	Y	Y
Manzanares-Leal GL, et al. 2022.	[65]	Y	Y	Y	Y	Y	Y	Y	Y
Zhang W, et al. 2024.	[85]	Y	Y	Y	Y	Y	N	Y	Y
Hu JT, et al. 2022.	[35]	Y	Y	Y	Y	N	N	Y	Y
Chao XP, et al. 2020.	[87]	Y	Y	Y	Y	Y	N	Y	Y
Chao XP, et al. 2021.	[86]	Y	Y	Y	Y	Y	N	Y	Y
Zeng M, et al. 2023.	[36]	Y	Y	Y	Y	Y	N	Y	Y
Logel M, et al. 2024.	[56]	Y	Y	Y	Y	Y	Y	Y	Y
Dou PN, et al. 2024.	[37]	Y	Y	Y	Y	Y	N	Y	Y
Lee YH, et al. 2020.	[57]	N	Y	Y	Y	Y	N	Y	Y
Cheng LQ, et al. 2020.	[41]	Y	Y	Y	Y	Y	Y	Y	Y
Zhang YC, et al. 2022.	[88]	Y	Y	Y	Y	N	N	Y	Y
Zeng WH, et al. 2023.	[89]	Y	Y	Y	Y	N	N	Y	Y
Ma YY, et al. 2023.	[90]	Y	Y	Y	Y	Y	N	Y	Y
McKee KS, et al. 2020.	[91]	Y	Y	Y	Y	Y	N	Y	Y
%		96.49	98.24	100.0	100.0	64.91	14.03	100.0	100.0

Appendix B 

**Table 4:** Critical appraisal of eligible cohort study.

Citation	Ref	Q1	Q2	Q3	Q4	Q5	Q6	Q7	Q8	Q9	Q10	Q11
Zhang HW, et al. 2018.	[59]	Y	N/A	Y	Y	N	N	Y	Y	Y	N/A	Y
Usyk M, et al. 2020.	[40]	Y	Y	Y	N	N	N	Y	Y	Y	N	Y
Camargo M, et al. 2022.	[42]	N/A	N/A	Y	Y	Y	N	Y	Y	Y	N/A	Y
Manzanares-Leal GL, et al. 2021.	[68]	N/A	N/A	Y	N	N	N	Y	Y	Y	N/A	Y
Wang WP, et al. 2023.	[43]	N/A	N/A	Y	N	N	N	Y	Y	Y	N/A	Y
Di Paola M, et al. 2017.	[44]	Y	Y	Y	Y	Y	N	Y	Y	Y	N/A	Y
Rodríguez SP, et al. 2022.	[45]	Y	N/A	Y	N	N	N	Y	Y	Y	N/A	Y
Wu RT, et al. 2019.	[46]	Y	Y	Y	Y	N	N	Y	Y	Y	U	Y
Shannon B, et al. 2017.	[47]	Y	Y	Y	Y	U	N	Y	Y	U	N/A	Y
Sims TT, et al. 2021.	[17]	Y	Y	Y	Y	N	N	Y	Y	U	U	Y
Zeber-Lubecka N, et al. 2022.	[66]	Y	Y	Y	Y	N	N	Y	Y	U	U	Y
Molina MA, et al. 2023.	[51]	N/A	N/A	Y	Y	N	N	Y	Y	Y	N/A	Y
Berggrund M, et al. 2020.	[48]	U	Y	Y	Y	Y	N	Y	Y	U	N/A	Y
Molina MA, et al. 2024.	[52]	Y	N/A	Y	Y	N	N	Y	Y	Y	N/A	Y
Wang Y, et al. 2024.	[67]	Y	Y	Y	Y	N	N	Y	Y	U	U	Y
Caselli E, et al. 2020.	[60]	N/A	N/A	Y	N	N	N	Y	Y	Y	Y	Y
Mitra A, et al. 2021.	[61]	Y	Y	Y	Y	N	N	Y	Y	Y	N/A	Y
Mitra A, et al. 2020.	[62]	N/A	N/A	Y	Y	N	N	Y	Y	Y	N	Y
Dong B, et al. 2022.	[50]	Y	N/A	Y	Y	N	N	Y	Y	Y	N/A	Y
D. I. Pierro F, et al. 2021.	[93]	N/A	Y	Y	Y	N	N	Y	Y	Y	N/A	Y
Shi WP, et al. 2022.	[49]	Y	Y	Y	Y	N	N	Y	Y	Y	N/A	Y
%		61.9	52.38	100.0	76.19	14.28	0.0	100.0	100.0	76.19	4.76	100.0

Appendix C 

**Table 5:** Critical appraisal of eligible randomized controlled trial.

Citation	Ref	Q1	Q2	Q3	Q4	Q5	Q6	Q7	Q8	Q9	Q10	Q11	Q12	Q13
Liu Y, et al. 2024.	[92]	Y	Y	Y	Y	Y	U	Y	Y	N	Y	Y	Y	Y
Ou YC, et al. 2019.	[94]	Y	Y	Y	Y	Y	Y	Y	Y	Y	Y	Y	Y	Y
Dellino M, et al. 2022.	[96]	Y	N	Y	N	N	N	Y	Y	N	Y	Y	Y	Y
Verhoevan, et al. 2012	[95]	N	N	Y	N	N	N	U	Y	NA	Y	Y	N	N
%		100.0	66.66	100.0	66.66	66.66	33.33	100.0	100.0	33.33	100.0	100.0	100.0	100.0
